# Supplementary material for: Vitamin D and Type 1 Diabetes Risk: A Systematic Review and Meta-Analysis of Genetic Evidence
Source: Nutrients. 2021 Nov 26;13(12):4260. doi: 10.3390/nu13124260 (PMC8707565; doi:10.3390/nu13124260)
Supplement: Supplementary file 1 [file nutrients-13-04260-s001.zip › NUTRIENTS VitD-T1D LN - Supplementary Materials 20211022.pdf]

Vitamin D and Type 1 Diabetes Risk: A Systematic Review and Meta-Analysis of Genetic Evidence

**Date:**	*22 October 2021*

**Project lead (corresponding author):**	*Liana Najjar* [a]<br>*liana.najjar@gmail.com*<br><br>*Bachelor of Clinical Exercise Physiology – Hons (Student)*
**Author List**	*Elina Hyppönen*[a]<br>*Professor in Nutritional and Genetic Epidemiology*<br>*Elina.Hypponen@unisa.edu.au*<br><br>*Ang Zhou*[a]<br>*Research Associate; PhD Medical Sciences*<br>*Ang.Zhou@unisa.edu.au*<br><br>*Joshua Sutherland*[a]<br>*BHSc Nut Med, Hons, PhD* Candidate,<br>*joshua.sutherland@mymail.unisa.edu.au*
**Affiliation, postal address:**	[a] *South Australian Health and Medical Research Institute (SAHMRI), Australian Centre for Precision Health, University of South Australia.*<br>*PO Box 11060*<br>*Adelaide 5001*<br>*South Australia*<br>*Australia*

Supplementary Materials

Figure Legend

Supplementary Figure S1. Funnel plot analysis for publication bias.

Supplementary Figure S2. Subgroup analysis of Caucasian participants for the association between selected genetic variants affecting serum 25-hydroxyvitamin concentrations and type 1 diabetes with the random effects model (coded by 25-hydroxyvitamin D increasing alleles). Squares represent the individual odds ratio estimate. Diamonds show the pooled effect. Horizontal bars represent the 95% confidence intervals.

Table Legend

Supplementary Table S1. Ovid: MEDLINE search strategy - the database coverage was 1946 to present, and the database was last searched on 1 April 2021.

Supplementary Table S2. Ovid: Embase Classic and Embase search strategy - the database coverage was 1947 to present, and the database was last searched on 1 April 2021.

Supplementary Table S3. Web of Science search strategy - the database coverage was 1947 to present, and the database was last searched on 1 April 2021.

Supplementary Table S4. OpenGWAS search strategy –annotated R script using R package. The database coverage was 2020 to present, and the database was last searched on 1 April 2021.

Supplementary Table S5. Quality assessment of the included studies: summary of critical appraisal using CASP tools.

Supplementary Material Author ICMJE Disclosure Forms.

Supplementary Table S1.

Ovid: MEDLINE search strategy - the database coverage was 1946 to present, and the database was last searched on 1 April 2021.

Set	Searches
#1	Humans/
#2	(Homo sapiens or Human).mp. [mp=title, abstract, original title, name of substance word, subject heading word, floating sub-heading word, keyword heading word, organism supplementary concept word, protocol supplementary concept word, rare disease supplementary concept word, unique identifier, synonyms]
#3	1 or 2
#4	Polymorphism, Single Nucleotide/
#5	(Nucleotide Polymorphism, Single or Nucleotide Polymorphism*, Single or Polymorphism*, Single Nucleotide or Single Nucleotide Polymorphism* or SNP* or Single Nucleotide Polymorphism).mp. [mp=title, abstract, original title, name of substance word, subject heading word, floating sub-heading word, keyword heading word, organism supplementary concept word, protocol supplementary concept word, rare disease supplementary concept word, unique identifier, synonyms]
#6	4 or 5
#7	Vitamin D/
#8	("25 hydroxyvitamin D" or "25(OH)D").mp. [mp=title, abstract, original title, name of substance word, subject heading word, floating sub-heading word, keyword heading word, organism supplementary concept word, protocol supplementary concept word, rare disease supplementary concept word, unique identifier, synonyms]
#9	7 or 8
#10	Genetic Variation/
#11	(Genetic Variation* or Variation*, Genetic or Diversity*, Genetic or Genetic Diversity* or Polymorphism*, Genetic* or Genetic Polymorphism* or mutation or "polymorphism*").mp. [mp=title, abstract, original title, name of substance word, subject heading word, floating sub-heading word, keyword heading word, organism supplementary concept word, protocol supplementary concept word, rare disease supplementary concept word, unique identifier, synonyms]
#12	10 or 11
#13	Diabetes Mellitus, Type 1/

#14	(Autoimmune Diabetes or Diabetes Mellitus, Insulin-Dependent or Diabetes Mellitus, Insulin-Dependent, 1 or Diabetes Mellitus, Juvenile-Onset or Diabetes Mellitus, Type I or Diabetes, Autoimmune or IDDM or Insulin-Dependent Diabetes Mellitus 1 or Juvenile-Onset Diabetes or Type 1 Diabetes or Type 1 Diabetes Mellitus).mp. [mp=title, abstract, original title, name of substance word, subject heading word, floating sub-heading word, keyword heading word, organism supplementary concept word, protocol supplementary concept word, rare disease supplementary concept word, unique identifier, synonyms]
#15	13 or 14
#16	6 or 12
#17	3 and 16
#18	9 and 17
#19	15 and 18

Supplementary Table S2.

Ovid: Embase Classic and Embase search strategy - the database coverage was 1947 to present, and the database was last searched on 1 April 2021.

Set	Searches
#1	Human/
#2	"Homo sapiens".mp. [mp=title, abstract, heading word, drug trade name, original title, device manufacturer, drug manufacturer, device trade name, keyword, floating subheading word, candidate term word]
#3	1 or 2
#4	Single Nucleotide Polymorphism/
#5	("Nucleotide Polymorphism, Single" or "Nucleotide Polymorphism*, Single" or "Polymorphism*, Single Nucleotide" or "Single Nucleotide Polymorphism*" or SNP* or "Single Nucleotide Polymorphism").mp. [mp=title, abstract, heading word, drug trade name, original title, device manufacturer, drug manufacturer, device trade name, keyword, floating subheading word, candidate term word]
#6	4 or 5
#7	Vitamin D/
#8	("25 hydroxyvitamin D" or "25(OH)D").mp. [mp=title, abstract, heading word, drug trade name, original title, device manufacturer, drug manufacturer, device trade name, keyword, floating subheading word, candidate term word]
#9	7 or 8
#10	Genetic Variation/
#11	("Genetic Variation*" or "Variation*, Genetic" or "Diversity*, Genetic" or "Genetic Diversity*" or "Polymorphism*, Genetic*" or "Genetic Polymorphism*" or mutation or "polymorphism*").mp. [mp=title, abstract, heading word, drug trade name, original title, device manufacturer, drug manufacturer, device trade name, keyword, floating subheading word, candidate term word]
#12	10 or 11
#13	Insulin dependent diabetes mellitus/
#14	("Autoimmune Diabetes" or "Diabetes Mellitus, Insulin-Dependent" or "Diabetes Mellitus, Insulin-Dependent, 1" or "Diabetes Mellitus, Juvenile-Onset" or "Diabetes Mellitus, Type 1" or "Diabetes, Autoimmune" or IDDM or "Insulin-Dependent Diabetes Mellitus 1" or "Juvenile-Onset Diabetes" or "Type 1 Diabetes" or "Type 1 Diabetes Mellitus").mp. [mp=title, abstract, heading word, drug trade name, original title, device manufacturer, drug manufacturer, device trade name, keyword, floating subheading word, candidate term word]

#15	13 or 14
#16	6 or 12
#17	3 and 16
#18	9 and 17
#19	15 and 18

Supplementary Table S3.

Web of Science search strategy - the database coverage was 1947 to present, and the database was last searched on 1 April 2021.

Set	Searches
#1	ALL=("Homo sapiens" OR "Human")<br>Indexes=SCI-EXPANDED, SSCI, A&HCI, CPCI-S, CPCI-SSH, ESCI, CCR-EXPANDED, IC Timespan=All years
#2	phism, Single Nucleotide" or "Nucleotide Polymorphism, Single" or "Nucleotide P olymorphism*, Single" or "Polymorphism*, Single Nucleotide" or "Single Nucleoti de Polymorphism*" or SNP* or "Single Nucleotide Polymorphism")<br>Indexes=SCI-EXPANDED, SSCI, A&HCI, CPCI-S, CPCI-SSH, ESCI, CCR-EXPANDED, IC Timespan=All years
#3	ALL=("Genetic variation*" OR "Variation*, genetic" OR "Variation, Genetic" OR "Diversit*, Genetic" OR "Genetic Diversit*" OR "Polymorphism*, Genetic" OR "G enetic Polymorphism*" OR "Polymorphism* Genetics" OR Mutation OR polymorp hism)<br>Indexes=SCI-EXPANDED, SSCI, A&HCI, CPCI-S, CPCI-SSH, ESCI, CCR-EXPANDED, IC Timespan=All years
#4	L=("Vitamin D" OR "25 hydroxyvitamin D" OR "25(OH)D")<br>Indexes=SCI-EXPANDED, SSCI, A&HCI, CPCI-S, CPCI-SSH, ESCI, CCR-EXPANDED, IC Timespan=All years
#5	ALL= ("Diabetes Mellitus, Type 1" OR "Autoimmune Diabetes" OR "Diabetes Mel litus, Insulin-Dependent" OR "Diabetes Mellitus, Insulin-Dependent, 1" OR "Diabetes Mellitus, Juvenile-Onset" OR "Diabetes Mellitus, Type I" OR "Diabetes, Autoimmune" OR "IDDM" OR "Insulin-Dependent Diabetes Mellitus 1" OR "Juvenile-Onset Diabetes" OR "Type 1 Diabetes" OR "Type 1 Diabetes Mellitus")<br>Indexes=SCI-EXPANDED, SSCI, A&HCI, CPCI-S, CPCI-SSH, ESCI, CCR-EXPANDED, IC Timespan=All years
#6	#3 or #2<br>Indexes=SCI-EXPANDED, SSCI, A&HCI, CPCI-S, CPCI-SSH, ESCI, CCR-EXPANDED, IC Timespan=All years
#7	*#6 AND #5 AND #4 AND #1*<br>*Indexes=SCI-EXPANDED, SSCI, A&HCI, CPCI-S, CPCI-SSH, ESCI, CCR-EXPANDED, IC Timespan=All years*

Supplementary Table S4.

OpenGWAS search strategy –annotated R script using R package. The database coverage was 2020 to present, and the database was last searched on 1 April 2021.

Set	Searches
#1	*# To query snp results in the openGWAS (15/01/2021):*<br>library(ieugwasr)
#2	*# meta-data for the gwas datasets in OpenGWAS database*<br>a <- gwasinfo()
#3	*# snp list*<br>snp <- c("rs12785878", "rs10741657", "rs117913124", "rs3755967", "rs17216707", "rs10745742", "rs8018720")
#4	*# gene list*<br>gene <- c("DHCR7/NADSYN1", "CYP2R1", "CYP2R1_lowfreq", "GC", "CYP24A1", "AMDHD1", "SEC23A")<br>gene <- data.frame(cbind(snp,gene))<br>colnames(gene) <- c("rsid","gene")
#5	*# gwas dataset list*<br>dataset <- a$id
#6	*# > length(dataset)*
#7	*# [1] 34513*
#8	*# to query snp-phenotype associations: batch the search: 1 search = 100 datasets; then loop over all the datasets listed in the OpenGWAS*<br>for(i in 1:ceiling(length(dataset)/100)) {<br>r <- associations(snp, dataset[(1+(i-1)*100):(100+(i-1)*100)], proxies = 1, r2 = 0.8, align_alleles = 1, palindromes = 1, maf_threshold = 0.3, access_token = check_access_token())<br>if(i==1) {r1=r} else r1=rbind(r1,r)
#9	*# to add dataset information to the results*<br>results <- merge(r1,a,by="id")
#10	*# to add gene information*<br>results2 <- merge(results,gene,by="rsid")<br>results <- results2
#11	*# to output the dataset*<br>write.table(results,"results location.txt", row.names=F, col.names=T, quote=F,sep="\t")

Supplementary Table S5.

Quality assessment of the included studies: summary of critical appraisal using CASP tools.

CASP Question	Are the results of the trial valid?	Were the cases recruited in an acceptable way?	Were the controls selected in an acceptable way?	Was the exposure accurately measured to minimise bias?	Have the authors taken account of the potential confounding factors in the design and/or in their analysis?	How precise are the results? (size of confidence intervals)	Overall quality
**Manousaki et al. 2021**	Met	Met	NA	Met	Met	Met	Low
**Almeida et al. 2020**	Partially met	Met	Met	Met	Met	Met	Low
**Nam et al. 2019**	Partially met	Met	Met	Met	Met	Met	Low
**Hussein et al. 2012**	Met	Met	Met	Met	Met	Partially met	Low
**Mahmoud et al. 2011**	Met	Met	Met	Met	Partially met	Met	Low
**Blanton et al. 2011**	Met	Partially met	Met	Met	Met	Met	Low
**Ramos-Lopez et al. 2007**	Met	Met	Met	Met	Met	Met	Low
**Klupa et al. 1999**	Met	Met	Met	Met	Partially met	Met	Low
**FinnGen**	NA	Met	NA	Met	Met	Met	Low
**UK Biobank**	NA	Met	NA	Met	Met	Met	Low

CASP Critical Appraisal Skills Program; NA not applicable.

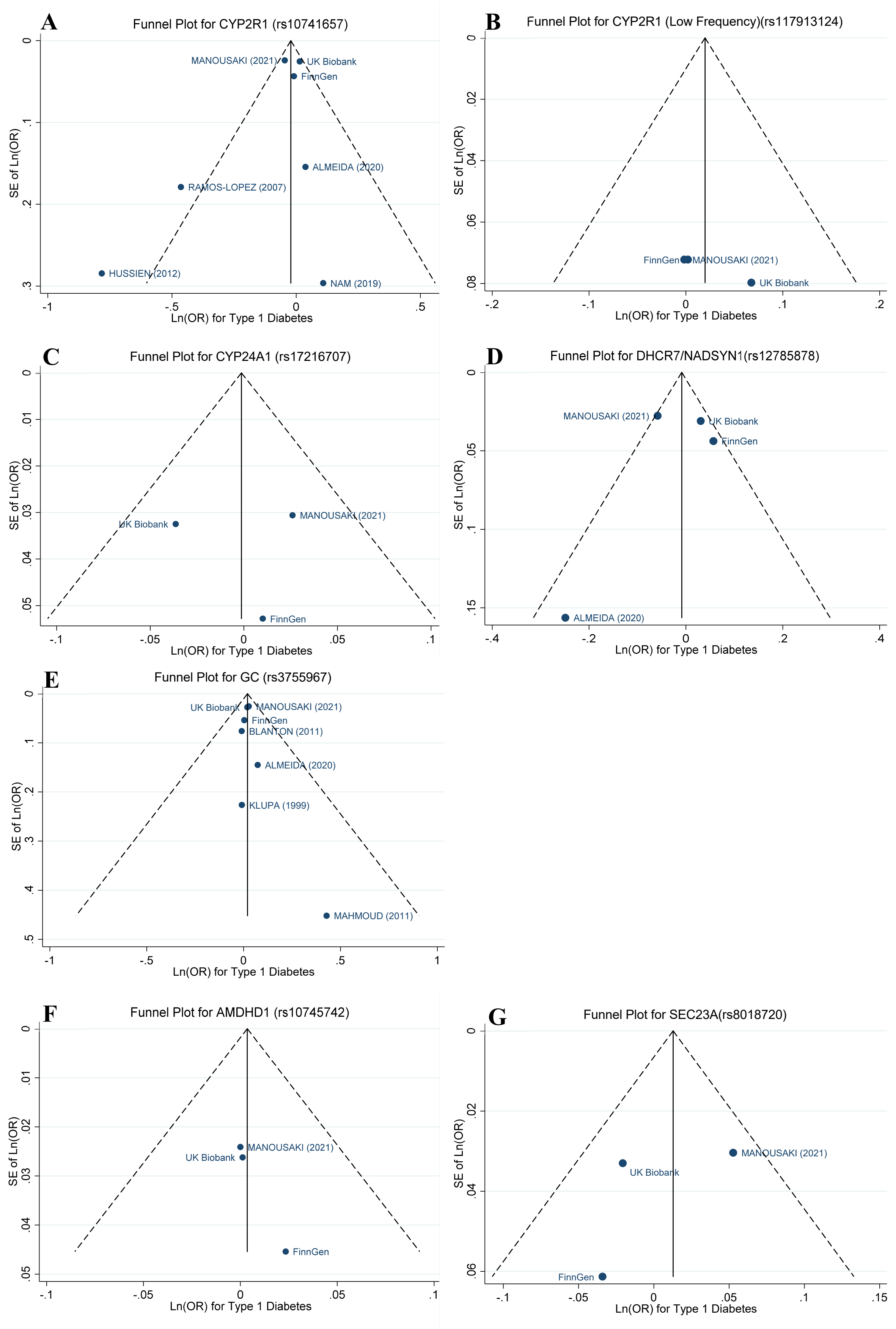

Supplementary Figure S1. Funnel plot analysis for publication bias in (A) *CYP2R1* rs10741657, (B) *CYP2R1* (low frequency) rs117913124*,* (C) *DHCR7/NADSYN1* rs12785878, (D) *GC* rs3755967, (E) CYP24A1 rs17216707*,* (F) *AMDHD1* rs10745742, (G) *SEC23A* rs8018720.

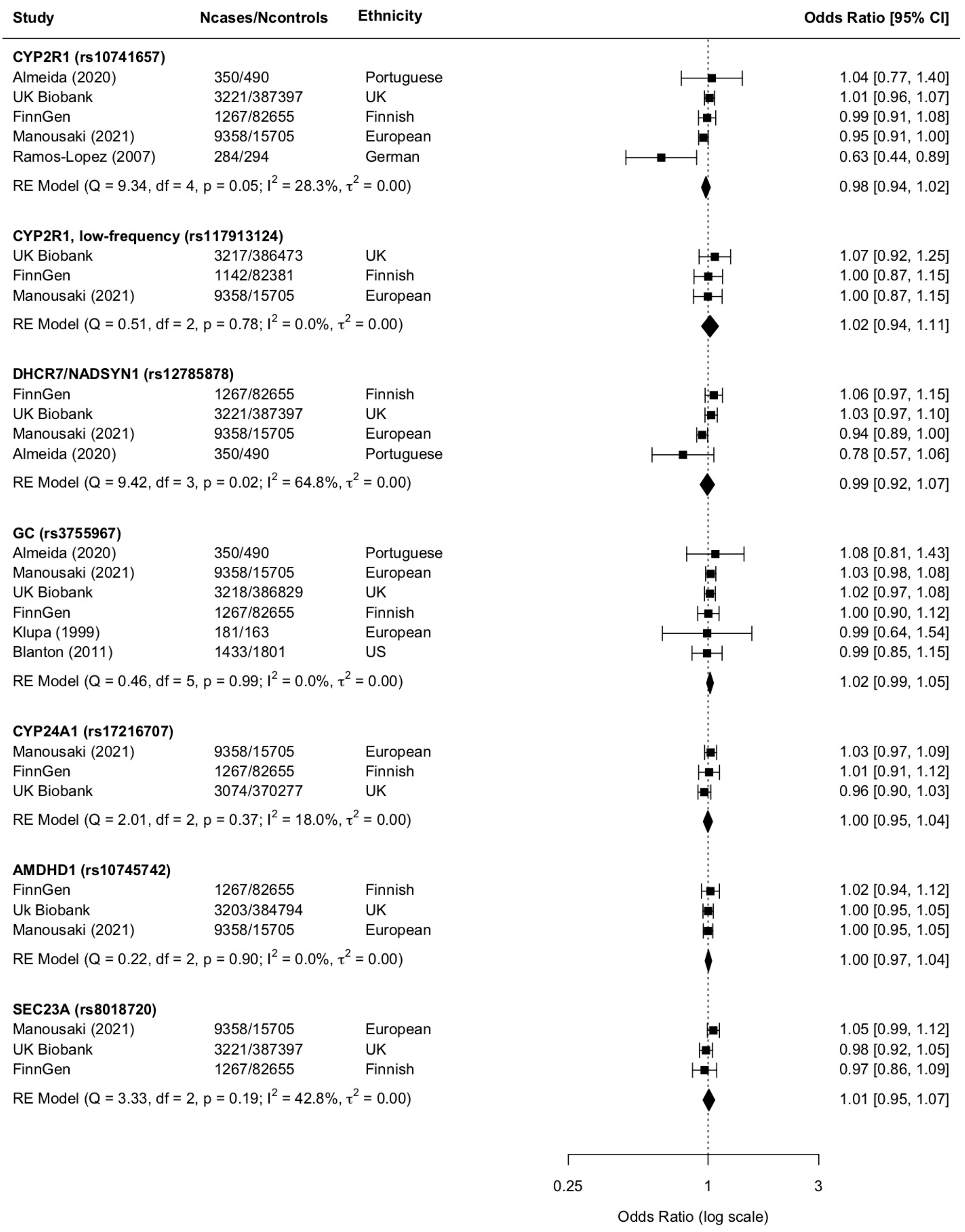

Supplementary Figure S2. Subgroup analysis of Caucasian participants for the association between selected genetic variants affecting serum 25-hydroxyvitamin concentrations (coded by 25-hydroxyvitamin D increasing alleles) and type 1 diabetes with the random effects model. Squares represent the individual odds ratio estimate. Diamonds show the pooled effect. Horizontal bars represent the 95% confidence intervals.

Supplementary Material Author ICMJE Disclosure Forms.

ICMJE DISCLOSURE FORM 1

Date: 26 July 2021

Your Name: Liana Najjar

Manuscript Title: Vitamin D and type 1 diabetes risk: A systematic review and meta-analysis of genetic evidence

Manuscript number (if known): NA.

In the interest of transparency, we ask you to disclose all relationships/activities/interests listed below that are related to the content of your manuscript. "Related" means any relation with for-profit or not-for-profit third parties whose interests may be affected by the content of the manuscript. Disclosure represents a commitment to transparency and does not necessarily indicate a bias. If you are in doubt about whether to list a relationship/activity/interest, it is preferable that you do so. The following questions apply to the author's relationships/activities/interests as they relate to the current manuscript only.

The author's relationships/activities/interests should be defined broadly. For example, if your manuscript pertains to the epidemiology of hypertension, you should declare all relationships with manufacturers of antihypertensive medication, even if that medication is not mentioned in the manuscript.

In item #1 below, report all support for the work reported in this manuscript without time limit. For all other items, the time frame for disclosure is the past 36 months.

		**Name all entities with whom you have this relationship or indicate none (add rows as needed)**	**Specifications/Comments (e.g., if payments were made to you or to your institution)**
		**Time frame: Since the initial planning of the work**	
1	All support for the present manuscript (e.g., funding, provision of study materials, medical writing, article processing charges, etc.)	____ None	

	**No time limit for this item.**		
**Time frame: past 36 months**			
2	Grants or contracts from any entity (if not indicated in item #1 above).	____ None	
3	Royalties or licenses	____ None	
4	Consulting fees	____ None	
5	Payment or honoraria for lectures, presentations, speakers bureaus, manuscript writing or educational events	____ None	
6	Payment for expert testimony	____ None	
7	Support for attending meetings and/or travel	____ None	
8	Patents planned, issued or pending	____ None	
9	Participation on a Data Safety Monitoring Board or Advisory Board	____ None	
10	Leadership or fiduciary role in other board, society, committee or advocacy group, paid or unpaid	____ None	
11	Stock or stock options	____ None	
12	Receipt of equipment, materials, drugs, medical writing, gifts or other services	____ None	
13	Other financial or non-financial interests	____ None	

Please place an "X" next to the following statement to indicate your agreement:

X___ I certify that I have answered every question and have not altered the wording of any of the questions on this form.

ICMJE DISCLOSURE FORM 2

Date: 22 July 2021

Your Name: Elina Hypponen

Manuscript Title: Vitamin D and type 1 diabetes risk: A systematic review and meta-analysis of genetic evidence

Manuscript number (if known): NA.

In the interest of transparency, we ask you to disclose all relationships/activities/interests listed below that are related to the content of your manuscript. "Related" means any relation with for-profit or not-for-profit third parties whose interests may be affected by the content of the manuscript. Disclosure represents a commitment to transparency and does not necessarily indicate a bias. If you are in doubt about whether to list a relationship/activity/interest, it is preferable that you do so. The following questions apply to the author's relationships/activities/interests as they relate to the current manuscript only.

The author's relationships/activities/interests should be defined broadly. For example, if your manuscript pertains to the epidemiology of hypertension, you should declare all relationships with manufacturers of antihypertensive medication, even if that medication is not mentioned in the manuscript.

In item #1 below, report all support for the work reported in this manuscript without time limit. For all other items, the time frame for disclosure is the past 36 months.

		**Name all entities with whom you have this relationship or indicate none (add rows as needed)**	**Specifications/Comments (e.g., if payments were made to you or to your institution)**
	**Time frame: Since the initial planning of the work**		
1	All support for the present manuscript (e.g., funding, provision of study materials, medical writing, article processing charges, etc.) **No time limit for this item.**	National Health and Medical Research Council	Competitive research funding, government supported
	**Time frame: past 36 months**		

2	Grants or contracts from any entity (if not indicated in item #1 above).	Tour de Cure<br>Medical Research Future Fund<br>Australian Research Council	Competitive research funding from the philanthropy (TdC) and government sources
3	Royalties or licenses	____ None	
4	Consulting fees	____ None	
5	Payment or honoraria for lectures, presentations, speakers bureaus, manuscript writing or educational events	____ None	
6	Payment for expert testimony	____ None	
7	Support for attending meetings and/or travel	____ None	
8	Patents planned, issued or pending	____ None	
9	Participation on a Data Safety Monitoring Board or Advisory Board	____ None	
10	Leadership or fiduciary role in other board, society, committee or advocacy group, paid or unpaid	____ None	
11	Stock or stock options	____ None	
12	Receipt of equipment, materials, drugs, medical writing, gifts or other services	____ None	
13	Other financial or non-financial interests	____ None	

Please place an "X" next to the following statement to indicate your agreement:
X___ I certify that I have answered every question and have not altered the wording of any of the questions on this form.

ICMJE DISCLOSURE FORM 3

Date: 26 July 2021

Your Name: Ang Zhou

Manuscript Title: Vitamin D and type 1 diabetes risk: A systematic review and meta-analysis of genetic evidence

Manuscript number (if known): NA.

In the interest of transparency, we ask you to disclose all relationships/activities/interests listed below that are related to the content of your manuscript. "Related" means any relation with for-profit or not-for-profit third parties whose interests may be affected by the content of the manuscript. Disclosure represents a commitment to transparency and does not necessarily indicate a bias. If you are in doubt about whether to list a relationship/activity/interest, it is preferable that you do so. The following questions apply to the author's relationships/activities/interests as they relate to the current manuscript only.

The author's relationships/activities/interests should be defined broadly. For example, if your manuscript pertains to the epidemiology of hypertension, you should declare all relationships with manufacturers of antihypertensive medication, even if that medication is not mentioned in the manuscript.

In item #1 below, report all support for the work reported in this manuscript without time limit. For all other items, the time frame for disclosure is the past 36 months.

		**Name all entities with whom you have this relationship or indicate none (add rows as needed)**	**Specifications/Comments (e.g., if payments were made to you or to your institution)**
**Time frame: Since the initial planning of the work**			
1	All support for the present manuscript (e.g., funding, provision of study materials, medical writing, article processing charges, etc.) **No time limit for this item.**	____ None	
**Time frame: past 36 months**			

2	Grants or contracts from any entity (if not indicated in item #1 above).	____ None	
3	Royalties or licenses	____ None	
4	Consulting fees	____ None	
5	Payment or honoraria for lectures, presentations, speakers bureaus, manuscript writing or educational events	____ None	
6	Payment for expert testimony	____ None	
7	Support for attending meetings and/or travel	____ None	
8	Patents planned, issued or pending	____ None	
9	Participation on a Data Safety Monitoring Board or Advisory Board	____ None	
1 0	Leadership or fiduciary role in other board, society, committee or advocacy group, paid or unpaid	____ None	
1 1	Stock or stock options	____ None	
12	Receipt of equipment, materials, drugs, medical writing, gifts or other services	____ None	
1 3	Other financial or non-financial interests	____ None	

Please place an "X" next to the following statement to indicate your agreement:

X___ I certify that I have answered every question and have not altered the wording of any of the questions on this form.

ICMJE DISCLOSURE FORM 4

Date: 26 July 2021

Your Name: Joshua Paul Sutherland

Manuscript Title: Vitamin D and type 1 diabetes risk: A systematic review and meta-analysis of genetic evidence

Manuscript number (if known): NA.

In the interest of transparency, we ask you to disclose all relationships/activities/interests listed below that are related to the content of your manuscript. "Related" means any relation with for-profit or not-for-profit third parties whose interests may be affected by the content of the manuscript. Disclosure represents a commitment to transparency and does not necessarily indicate a bias. If you are in doubt about whether to list a relationship/activity/interest, it is preferable that you do so. The following questions apply to the author's relationships/activities/interests as they relate to the current manuscript only.

The author's relationships/activities/interests should be defined broadly. For example, if your manuscript pertains to the epidemiology of hypertension, you should declare all relationships with manufacturers of antihypertensive medication, even if that medication is not mentioned in the manuscript.

In item #1 below, report all support for the work reported in this manuscript without time limit. For all other items, the time frame for disclosure is the past 36 months.

		**Name all entities with whom you have this relationship or indicate none (add rows as needed)**	**Specifications/Comments (e.g., if payments were made to you or to your institution)**
**Time frame: Since the initial planning of the work**			
1	All support for the present manuscript (e.g., funding, provision of study materials, medical writing, article processing charges, etc.) **No time limit for this item.**	____ None	
**Time frame: past 36 months**			

2	Grants or contracts from any entity (if not indicated in item #1 above).	____ None	
3	Royalties or licenses	____ None	
4	Consulting fees	____ None	
5	Payment or honoraria for lectures, presentations, speakers bureaus, manuscript writing or educational events	____ None	
6	Payment for expert testimony	____ None	
7	Support for attending meetings and/or travel	____ None	
8	Patents planned, issued or pending	____ None	
9	Participation on a Data Safety Monitoring Board or Advisory Board	____ None	
10	Leadership or fiduciary role in other board, society, committee or advocacy group, paid or unpaid	____ None	
11	Stock or stock options	____ None	
12	Receipt of equipment, materials, drugs, medical writing, gifts or other services	____ None	
13	Other financial or non-financial interests	____ None	

Please place an "X" next to the following statement to indicate your agreement:

X___ I certify that I have answered every question and have not altered the wording of any of the questions on this form.
